# A Review of Immunomodulatory Reprogramming by Probiotics in Combating Chronic and Acute Diabetic Foot Ulcers (DFUs)

**DOI:** 10.3390/pharmaceutics14112436

**Published:** 2022-11-10

**Authors:** Prakhar Srivastava, Tesalonika Sondak, Karthikeyan Sivashanmugam, Kwang-sun Kim

**Affiliations:** 1 Department of Chemistry and Chemistry Institute of Functional Materials, Pusan National University, Busan 46241, Korea; 2 School of Biosciences and Technology, High Throughput Screening Lab, Vellore Institute of Technology, Vellore 632014, Tamil Nadu, India

**Keywords:** diabetic foot ulcer, biofilm, acute, chronic, cytokines, macrophages, probiotics

## Abstract

Diabetic foot ulcers (DFUs) are characterized by a lack of angiogenesis and distal limb diabetic neuropathy. This makes it possible for opportunistic pathogens to protect the biofilm-encased micro-communities, causing a delay in wound healing. The acute and chronic phases of DFU-associated infections are distinguished by the differential expression of innate proinflammatory cytokines and tumor necrosis factors (TNF-α and -β). Efforts are being made to reduce the microbial bioburden of wounds by using therapies such as debridement, hyperbaric oxygen therapy, shock wave therapy, and empirical antibiotic treatment. However, the constant evolution of pathogens limits the effectiveness of these therapies. In the wound-healing process, continuous homeostasis and remodeling processes by commensal microbes undoubtedly provide a protective barrier against diverse pathogens. Among commensal microbes, probiotics are beneficial microbes that should be administered orally or topically to regulate gut–skin interaction and to activate inflammation and proinflammatory cytokine production. The goal of this review is to bridge the gap between the role of probiotics in managing the innate immune response and the function of proinflammatory mediators in diabetic wound healing. We also highlight probiotic encapsulation or nanoformulations with prebiotics and extracellular vesicles (EVs) as innovative ways to tackle target DFUs.

## 1. Introduction

The term “neuropathy” in diabetes is a generic term that refers to the loss of sensation and steadiness of the distal limb. The loss of sensation affects peripheral blood movement. Therefore, dysregulating the protective sensation leads to systemic inflammatory status of diabetic foot ulcers (DFUs) [1]. Characterizing the type of wound—which considers various factors such as the length of the wound, the size of the ulcer, and its location—is a crucial step in the evaluation of DFUs. These parameters primarily indicate whether the ulcer is acute or chronic in nature [2]. From the acute to the gangrene stages, DFUs express differently. The acute stage begins with a recurring loss of sensation and subcutaneous hemorrhage of the lesion, which usually goes unnoticed and results in foot deformity. It normally heals in three weeks, but improper wound management in the acute stage leads to a chronic or infectious stage with inflammatory dysregulation [3]. 

However, due to the broad pathophysiology of DFU infection, relying on any single therapy [4] causes local inflammation, bleeding, and a high increase in blood oxygen levels. To overcome such limitations, therapies targeting immune cells using fibroblasts, stem cells, grafts, monoclonal antibodies, and bioactive molecules have been recently developed. However, these therapies have some limitations. A short half-life, the need for repeated administration, high costs of production, short-term bioactivity, insufficient data on large populations, limited downstream processing, renal clearance, and ineffectiveness against polymicrobial infections are all issues that must be addressed [5].

Gut immunological homeostasis, which is maintained by the interaction among intestinal microflora, is crucial in controlling the host’s inflammatory response. The gut microbiota influences various parts of the human body, including the brain, liver, and skin. Concerning DFU infection, commensal microbes (*Staphylococcus epidermidis*, *Propionibacterium*, and *Cutibacterium* species) on human skin have direct coordination with the gut microbiota and govern chronic responses during skin–pathogen contact [6]. Among gut microbiota, probiotics have been used to treat a variety of health issues since long ago and have also shown promising results as immunomodulators during chronic illness [7]. Probiotics already have several advantages, including non-toxicity, user-friendliness, strong immunity, a longer half-life, an easy route of administration, and simple downstreaming [8]. However, questions still remain about the use of probiotics as a DFU therapeutic. Constant remodeling of probiotics through encapsulation or nanoformulation using prebiotics and synbiotics, biogenic nanoparticles (NPs), and extracellular vesicle (EVs) originating from probiotics can open new avenues in the field of DFU management. These strategies are still unexplored.

In this review, we first introduce the importance of the gut–skin axis in maintaining skin homeostasis and emphasize it as a new route for DFU therapy using probiotics. Then, the role of probiotics in maintaining acute and chronic conditions of DFUs will be further discussed. Further, a novel idea on how probiotics can reprogram acute and chronic modulation during DFU infections will be introduced as a future application of probiotics. These include cutting-edge technologies such as the encapsulation or nanoformulation of probiotics using prebiotics and synbiotics, biogenic NPs, and EVs. Overall, our review will provide perspectives on probiotic-mediated therapeutics against infections in acute and chronic DFUs.

## 2. Method

For this review paper, we searched the databases (PubMed, Science Direct, Web of Science, and Scopus) for articles that majorly dealt with the following keywords: “diabetic foot ulcer”, “diabetes”, “biofilm”, “acute”, “chronic”, “pathogen”, “inflammatory cytokine”, “cytokine”, “macrophage”, “fibroblast growth factor”, “stem cell”, “neuropeptide”, “monoclonal antibody”, “ bioactive”, “gut skin axis”, “probiotic”, “symbiotic”, “extracellular vesicle”, “postbiotic”, and “paraprobiotics”. In addition, case studies, review studies, and retrospective studies involving randomized clinical trials relevant to diabetic foot ulcer and its therapeutic approaches were included. Moreover, we explored the bibliographies of all retrieved and relevant articles.

## 3. Diabetes: A Constant Fluctuation in Blood Glucose Levels

According to the International Diabetes Federation Atlas (IDFA), diabetes is prominent in every part of the world. Over 537 million people suffered from diabetes around the world in 2019, and cases are expected to increase to 700–750 million worldwide by 2045 [9]. A global survey revealed 6.7 million deaths due to diabetes in 2021 (1 every 5 s) involving demographic locations including Africa (416,000), Europe (1.1 million), the Middle East (796,000), Southeast Asia (747,000), and the Western Pacific (2.3 million). Diabetes in the current scenario holds the position of being a chronic and long-term hypo/hyperglycemic condition that can result in multiple complications such as cardiovascular disorders, neuropathy, nephropathy, and retinopathy [10]. The lifestyle pattern and genetic disposition from different demographic locations normally explain the prognosis of diabetes, but substantial research indicated that the gut microbiota, which are crucial to human physiology, metabolism, and dysbiosis, have a direct role in type 2 diabetes mellitus (T2DM) [11]. T2DM is frequently associated with obesity; it is noteworthy that certain compositions of gut microbiota have been linked to insulin resistance and glycemic control problems [12]. Changes in the composition of gut bacteria may contribute to the etiology of diseases such as obesity, diabetes, and kidney disease, the prevalence of which are still rising globally [13]. These situations generally involve diabetic ketoacidosis and hyperosmolar state, which play a significant role in automatic and peripheral neuropathy [14]. The glycemic complications related to diabetes involve both hypoglycemia and hyperglycemia, but hyperglycemic DFUs account for most of the mortality and morbidity [15]. 

## 4. The Diabetic Foot Ulcer: A Long-Lasting Foot Deformity

The DFU, one of the major complications of diabetes, affects 25 to 30% of people during their lifetime. Among them, 20% undergo lower limb amputation with an annual mortality rate of 11% for DFUs and 22% for amputees [16]. Every 20 to 30 s, a lower limb gets amputated, with DFUs accounting for 85–95% of the cases [17]. Patients with high blood glucose levels exhibited severe pathological conditions such as tissue hypoxia, leading to inadequate blood supply to the vascular endothelial cells [18]. The prognosis involves a localized injury to the distal portion of the ankle, which goes unnoticed due to inefficient sensory response, peripheral neuropathy, and vascular lesions. The lesion paves the way for opportunistic pathogens that increase the chances of tissue hypoxia by reducing the process of angiogenesis and endothelial revascularization, which in turn delay the wound-healing process. This delay in the wound-healing process results in the chronic stages of DFUs and the formation of gangrene [19,20]. The process from neuropathy to gangrene follows a gradual infectious route, which leads to a high microbial bioburden [21] and delays the wound-healing process at chronic stages due to the formation of microbial communities known as biofilms. High blood glucose levels increase the concentration of proteins and carbohydrates, which form the basic core for persistent infections. About 40 to 80% of diabetic patients develop recurrent infections, and between 20 to 25% develop deep infections with osteomyelitis [22]. To select the appropriate treatment course and manage care for DFU patients, several classification systems have been developed to elucidate the characteristics of DFUs and the severity of infections as described below. 

### 4.1. Evaluation and Classification of DFU Extent

The systematic evaluation of DFUs defines the constant prognosis of foot ulcers. Health professionals determine the etiology of the foot and verify the extent of lesions that involve acute Charcot foot or chronic ischemic foot [23]. Various classification systems have been developed to describe ulcer characteristics and its lower limb extremities.

#### 4.1.1. Meggitt–Wagner (MW) Classification System

Developed in the 1970s, this system comprises six ulcer grades that range from 0 to 5. This system assesses the pro-ulcerative stage, superficial infection, subcutaneous infection, deep ulcer in tendons, forefoot gangrene, and whole-foot gangrene (50% foot infection) [24]. This system is simple and widely accepted for predicting lower extremity amputation. However, this system is not recommended for use in assessments of DFUs because it does not adequately address all DFU subtypes and the spectrum of infections. The major limitations of this system are the infection rate and tissue viability [25].

#### 4.1.2. University of Texas (UT) Classification

This system is a modified version of the MW classification that has also been effective in predicting lower extremity amputation. Moreover, this system overcomes all of the shortcomings of MW, especially the depth of lesion and infection rate [26]. This system uses four grades (0 to 3; depth) and four stages (A–D; severity of wound) to classify DFUs by marking the presence of infection, ischemia, or both [27]. This system helps predict the infection rate and the rate of amputation and is used in practice; however, it is ineffective in determining the degree of neuropathy and microbial load differentiation.

#### 4.1.3. Perfusion, Extent, Depth, Infection, and Sensation (PEDIS)

This system was developed in 2003 by International Working group of Diabetic Foot (IWGDF) solely for determining the rate of infection. It includes four categories. The first is uninfected or pro-ulcerative (the same as MW and UT); the second involves signs of infection, erythema, or colitis. The last two stages involve the severity of the infection and its related circumstances such as moderate to severe infection and systemic toxicity involving fever, chills, hypertension, and cardiovascular disorders [28]. It is a complex system that does not truly define the ulcer types but has an advantage in terms of choosing empirical antibiotic therapy against chronic infection.

#### 4.1.4. Saint Elian Wound Score System (SEWSS)

This is an advanced version of the PEDIS system. This system uses three grades (I to III) based on 10 factors in the following categories: location, topographic aspects, the number of affected zones, ischemia, infection, edema, neuropathy, depth, area, and wound-healing phase. The total score is 6 to 30 points, and the score can switch to a grade. It is an advantageous system compared to others and can efficiently detect the outcome of DFUs (minor amputation and wound healing), but it is time-consuming [29].

#### 4.1.5. Site, Ischemia, Neuropathy, Bacterial Infection, Area, and Depth (SINBAD)

This is a versatile and rapid system that uses five clinical features (site of infection, ischemia condition, neuropathy, bacterial infection, and depth), which are graded as either present (0) or absent (1). This system is primarily used in various rural regions where DFU occurrence is very prominent and does not require any medical setup for evaluation. SINBAD generally provides a high degree of versatility and also helps to differentiate between the acute and chronic conditions in the context of validating research and consistent results [30].

## 5. Timeline and Stages of DFUs

**Figure 1:**
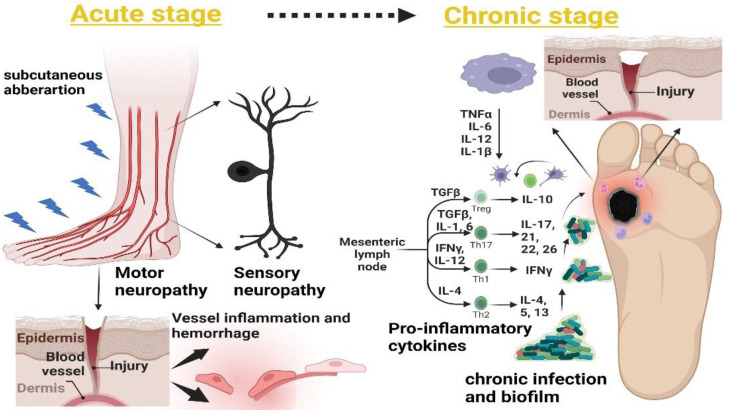
Acute and chronic conditions of the DFU. The acute stage includes subcutaneous aberration, which is associated with loss of sensation at the distal part of the wound. The continuous aberration at the wound causes blood vessel angiogenesis to fail, resulting in inflammation at the wound site (**left**). The chronic stage is characterized by continuous wound damage and the movement of proinflammatory responses. This causes skin moistening, which allows opportunistic pathogens to form a biofilm, delaying the wound-healing mechanism during a DFU (**right**).

The open lesion and complete vascularization take 8 to 14 days for DFU wound closure [31], but the normal healing process depends on the stages of the foot ulcer, considering the previous classification of DFUs (Section 4.1) from the superficial wound stage to gangrene of whole foot. A wound can be defined as an acute or chronic type. The latter is the most infectious and non-healing condition and involves factors such as high infection and release of proinflammatory responses. The major pathways in acute and chronic conditions are shown in Figure 1.

### 5.1. Sensory and Motor Neuropathy

Sensory and motor neuropathy involve constant pressure and local necrosis. The high pressure on the peripheral veins leads to constant injuries that result in autonomic tissue injury. There might be instances that can result in small areas of gangrene or ulceration [32]. Additionally, autonomic neuropathy includes thermoregulatory dysregulation that involves excessive sweating, cracking, and edema in the foot region, which further adds to the ulceration [33].

### 5.2. Subcutaneous Hemorrhage

The skin breakdown during the tissue damage leads to subcutaneous hematoma involving deep tissue cellulitis near bones and tendons, which is normally manifested as pain and tenderness of the epidermis and dermis [34]. The loss of sensory and motor reflexes results in the formation of thickened skin that is generally referred to as a “callus”, which causes constant pressure that leads to foot deformity. The formation of small lesions near the callus region can be treated by using debridement and fitting shoes, but improper management can subsequently lead to foot deformation and mechanical stress [35].

### 5.3. Foot Deformity

Various risk factors such as deep tissue necrosis and motor and sensory polyneuropathy lead to diabetic neuro-osteoarthropathy, a term related to deformity in the tarsus and metatarsus region of the foot. The classic example is Charcot foot associated with the disorientation of bones and joints, which are further related to bone resorption and site-specific inflammation [36]. A study conducted in a Copenhagen wound-healing center from 1995 to 2015 showed a significant relationship between acute Charcot foot and the prognosis of ulcer. This study highlighted the social economic impact of Charcot foot ulcers on type I diabetic patients. Out of 173 patients, only 4% were able to perform their normal routine jobs, while 60–65% were below the basic social economic conditions [37]. 

### 5.4. Acute Inflammatory Responses (Neutrophils and Macrophages) and Accumulation of Extracellular Matrix (ECM)

A hyperglycemic condition always shows a low-grade inflammatory response to the microvascular environment during DFUs. The increased diabetic stress results in high inflammatory responses that result in the release of white blood cells (WBS) and acute phase proteins including C-reactive proteins, fibrinogen, chemokines, and interferon-γ [38]. A high glycemic index and localized inflammation result in the production of interleukins such as IL-6 from the macrophage. IL-6 is known for its proinflammatory activities; its constitutive expression results in a delay in wound healing [39]. Muller et al. [40] showed the effects on type 2 diabetic patients of an increased release of IL-6 and tumor necrosis factor (TNF) receptors, highlighting the role of IL-6 modulation as a diagnostic marker in the evaluation of inflammatory response during DFUs. Localized inflammation is related to innate and proinflammatory responses during chronic conditions, including a higher count of neutrophils, T cells, and mast cells, which represent the markers and generally delay the wound-healing process. The high expression of neutrophils is further related to revascularization via protease production, collagen, cathepsin, and matrix-degrading substances such as metalloproteinase [41].

### 5.5. Abnormal Matrix Metalloproteinase (MMP) Expression

Matrix metalloproteinases are important factors in wound healing. The expression of MMPs regulates the inflammatory phase and the endothelial basement for angiogenesis, but in cases of chronic DFU, the abnormal expression of MMP-9 significantly delays the wound-healing process [42]. The overexpression of MMP-9 upregulates the protease production, which results in the accumulation of macrophages and neutrophils, which in turn results in excessive collagen production that hampers the normal wound-healing process [43].

### 5.6. Bacterial Invasion and Persistent Infection

The overall prognosis of a DFU lies in the management of foot ulcers, but the bottom line of the management depends on the severity of the DFU. Inadequate management during sensory and motor neuropathy and constant friction of the foot leads to the moistening of the foot region that involves the movement of opportunistic pathogens. A total of 80% of traumatic wounds lead to amputation; the remaining 20% lead to recurrent bacterial infection [44]. Inflammation and persistent infection, which are the major causes of amputation, involve constant chemotaxis movement of T and B cells during infection. Infection is the only factor in DFUs that involves both the acute and chronic stages of DFUs; i.e., ischemia, callus formation, and hyperproduction of the proinflammation cytokine [45].

## 6. Microbial Bioburden in DFUs

The microbial spectra in DFUs are often related to the moistness and breaking of the cutaneous region of the wound, which in due course adversely affect the normal healing of the wound [46]. Normally, the onset of foot infections involves superficial or subcutaneous categories, but these infections can spread to the tendons, joints, and bones, causing severe osteomyelitis [47]. The microbial diversification varies according to the extent of the DFU infection. According to the guidelines of the Infectious Disease Society of America (IDSA), superficial infections generally involve aerobic microorganisms such as *Staphylococcus*, *Micrococcus*, *Corynebacterium*, *Pseudomonas*, and *Klebsiella* spp. [45]. A deep tissue or osteomyelitis condition involves the presence of some facultative anaerobic and strictly anaerobic microorganisms including *Peptostreptococcus* spp., *Bacteroides* spp., *Prevotella*, *Fusobacterium*, and *Pseudomonas* spp. [48]. The heterogeneous nature of wounds involves polymicrobial interaction, which corresponds to the high microbial load during chronic infection [49]. However, Gram-negative bacteria have a prevailing existence in the wound microbiome compared to Gram-positive bacteria. The various microbial communities coexisting in DFUs play different roles and are therefore essential in the multiple stages of DFUs. Chronic wounds often involve various small molecules or autoinducers that generally act as biomarkers in metagenomic studies of wounds [50]. Polymicrobial infections often involve cell-to-cell communication via these autoinducers, which enables the production of virulence factors such as the protease, elastase, rhamnolipid, and extracellular polysaccharide matrix (EPS) [51]. The synergistic adaptation of microbes to the wound site includes embedded micro-communities in a polymeric matrix known as a biofilm. The term biofilm is often related to the chronicity of a wound; i.e., the higher the microbial load, the more compact the structure of the biofilm will be [52]. The presence of a biofilm in DFU cases involves 60 to 80% of chronic cases and 8 to 10% of acute cases. These micro-communities strongly limit the entry of antimicrobial agents to the site of infection, resulting in prolonged hospitalization [53]. Aerobic microbes such as *Pseudomonas* and *Staphylococcus* are generally frequently isolated from chronic infection sites. These microbes also produce strong biofilms in the polymicrobial load that influence the presence of other pathogens [54]. A study conducted on *Pseudomonas* and *Staphylococcus* biofilms showed the production of virulence factors such as exopolysaccharide (EPS), showcasing the prolonged chronic nature of the wound. The increased microbial burden also showed the accumulation of inflammatory responses that affected the normal healing process [55]. The persistent infection response involves the movement of innate immune cells such as neutrophils, macrophages, and leukocytes. This ultimately increases the reactive oxygen species (ROS) production and release of proinflammatory markers at the site of infection, leading to a non-healing situation of the ulcer [56]. 

## 7. Innate Immune and Proinflammatory Mediators in Non-Healing DFUs

Chronic diabetic wounds have four distinct phases of healing: (i) the inflammatory response, (ii) the proliferative stage, (iii) migration, and (iv) revascularization [57]. In acute conditions, motor and sensory neuropathy are generally addressed by proper management and wound assessment, which involve evaluation of subcutaneous hemorrhage and deformity. The chronic condition involves immune outburst at the site of infection and an increased expression of innate immune cells such as mast cells, lymphocytes, neutrophils, macrophages, C-reactive proteins, proinflammatory cytokines, and ROS, which cause a delay in re-epithelization and angiogenesis [58].

### 7.1. Innate Immune Cells

Innate immune cells including neutrophils and macrophages are primary cells involved in the wound-healing process and early vascularization of a wound [59]. However, during a non-healing diabetic wound, there is a slow chemotaxis movement of cells that results in decreased adherence and proliferation, leading to an inadequate phagocytic elimination [60]. Apart from neutrophils, macrophages have also shown an impaired role during the wound-healing process. Aberrant stimulation of macrophages during diabetic wounds involves restricted switching from M1 to M2 macrophages, resulting in a constant outburst of IL-1β and TNF-α [61]. A study conducted on diabetes-induced mice showed an elevated level of a proinflammatory cytokine (IL-1β) that was exhibited by the proinflammatory responses of altered M1 and M2 macrophages at the site of infection. This differential expression of transforming growth factors (TGFs), an insulin-like growth factor (IGF-1), and MMP-9 highlighted the impaired response of macrophage switching in delaying the wound-healing process [62]. Macrophages and monocytes also play important roles in wound healing and collagen deposition during epithelization. Impaired regulation of either of the two leads to the aberrant transition of proinflammatory cytokines, resulting in delayed wound healing [63]. 

### 7.2. Proinflammatory Cytokines

ILs and TNF generally constitute the major classes of proinflammatory cytokines that promote insulin resistance and impair healing. Kolumam et al. [64] showed that IL-6, -18, -22, and -24 are highly expressed in diabetic wounds compared to non-diabetic wounds, highlighting the differential expression of pro- and anti-inflammatory responses during DFUs. Similarly, TNF-β is generated by monocytes and macrophages and helps to induce T- and B-cell responses to regulate the vascularization of an endothelial cell. During DFU dysregulation, an immunomodulatory burst (IL-1β and TNF-α) results in an impaired insulin metabolism, altering the humoral expression (B-cell regulation) [65]. A study conducted by Trengove et al. [66] on patients from Western Australian hospitals with chronic non-healing venous ulcers highlighted the direct relationship of an altered innate response and altered wound healing. The bioassay showed higher expressions of IL-1 and -6. Furthermore, levels of IL-1α, IL-β, and TNF-α showed a marked increase in the non-healing wound. Similarly, growth factors such as epidermal growth factor (EGF), platelet-derived growth factor (PDGF), and TGF-β1 were also altered in chronic venous ulcers.

Apart from innate immune cells, TLRs are one set of immune cells that link innate immunity with adaptive immunity and play a role against microbial invasion by targeting the lipopolysaccharide layer of pathogens [67]. The significant expression of TLRs during infection results in the production of proinflammatory responses; normally, during the healing of a wound, the expression of TLRs (TLR-2 and -4) decreases with tissue vascularization. A study by Pukstad et al. [68] on non-healing chronic wound ulcers showed a significant increase in the expression of TLR-2 and -4, which constitutively led to the expression of proinflammatory markers (IL-1α, IL-1β, and MIP-1δ). Hence, reducing the levels of anti-inflammatory cytokines (IL-8 and MIP-1α) and dysregulating TLRs can modulate the healing procedure in chronic wound infections.

### 7.3. Matrix Metalloproteinase

Apart from proinflammatory cytokines, MMPs also exert inflammatory responses in chronic diabetic wounds. In recent years, MMPs have gained a higher value in studies related to non-healing diabetic wounds and have been linked to the impaired activity of IL-1β and TNF-α [69]. A study conducted on acute and chronic surgical wounds showed a significantly higher expression of MMP-9 in chronic as compared to acute wounds. The increased expression of MMP-9 (from 10- to 25-fold) also highlighted the reduced expression of elastase, which was directly correlated with the delay in the endothelial vascularization [70]. MMPs play a vital role in tissue remodeling, and their differential expression (MMP-1, -2, -8, and -9) regulates poor wound healing [71].

### 7.4. Toll-Like Receptors (TLRs)

Impaired wound healing during DFUs involves the activation of TLRs, which recognize inflammatory factors such as pathogen-associated molecular patterns (PAMPs) of bacteria during chronic DFUs [72]. PAMPs generally participate in the activation of TLR signaling and have an important role in infection-mediated immune responses in DFUs [73]. Alteration in the microbial bioburden results in the expression of differential TLRs (TLR-1, -2, -4, -6, and -8); these factors have also been used as inflammatory biomarkers in DFUs. A study by Dasu et al. [74] on streptozotocin-induced mice showed increased expression of TLR-2 and TLR-4 with a high glycemic index, highlighting the T-cell-mediated responses in the inflammatory phase of DFUs. Creely et al. [75] also showed that the hyperglycemic state in DFUs results in inflammation in adipose tissues that increased the expression of TLR-2 and TLR-4. This in turn increased the expression of proinflammatory cytokines (TNF-α, IL-6, and NF-kB), impairing the normal healing process. EPS formation and the biofilm matrix also stimulate the production of proinflammatory cytokines (TLRs and TNF-α). A study on biofilm-producing *S. aureus*-induced wounds on a type 2 diabetic rat model showed an increased outburst of TLR-2, 4, and TNF-α, supporting a direct correlation with biofilm-mediated inflammatory responses [76]. DNA methylation also has a significant role in altering the normal wound-healing procedure. A study by Singh et al. [77] on the epigenetic role of TLR-2 showed a significant downregulation of TLR-2 in type 2 diabetes, highlighting the partial methylation of the CpG islands in the chronic diabetic wound.

## 8. Novel Therapies Targeting Inflammatory Modulators during Non-Healing DFUs

Based on our review, we understand that the multidisciplinary approaches of different specialties are required to decrease wound bioburdens and the chances of lower limb amputations. Since the management of DFU wounds varies from stage to stage [78], providing adequate care to the foot region is important. Conventional therapies such as debridement [79], hyperbaric oxygen therapy [80], shock wave therapy [81], offloading [82], larval therapy [83], laser therapy [84], and empirical antibiotic treatment [85] are promising as efficient treatments, but they are limited in terms of scope with respect to tissue vascularization and the healing process of ischemic ulcers. Therefore, the cost-to-benefit ratios are lower, and retrospective studies conducted on these therapies require more evidence from a larger demographic area. The major advantages and disadvantages of the above therapies are summarized in Table 1.

During diabetic wounds, it is imperative to reduce the production of proinflammatory cells and ROS. The delay in apoptosis results in tissue granularization and long-term chronic inflammation, which further contributes to the poor healing of the wound site, resulting in a loss of epithelization [93]. The previous alternatives shown in Table 1 have limitations in being used for a longer period; therefore, some novel therapies are being investigated that involve fibroblast growth factors, macrophage-regulating drug therapy, stem cell therapy, neuropeptides, MMP inhibitors, monoclonal antibodies, and plant-derived bioactive compounds. 

### 8.1. Fibroblast Growth Factor (FGF)-Associated Therapy

Basic fibroblast growth factors (bFGFs) have a prominent role in diabetic wound healing. They were found to activate signaling pathways of MAPK and JAK-STAT, which affect the basic movement of fibroblasts and keratinocytes in endothelial vascularization and collagen formation [94]. Therapies including fibroblasts have been used since the early 1990s because fibroblasts were regarded as a prominent factor in the formation of granular tissues during epithelization. A study on FGF and a keratinocyte-based hydrogel against diabetic rates showed a rapid healing rate and granularization of endothelial tissue. This study also showed that the fibroblast-associated hydrogel was effective against proinflammatory cytokines. It decreased IL-6, TNF-α, and IL-10 production and increased the expression of pro-healing factors such as TGF-β and collagen III after 1 to 2 weeks of therapy [42]. 

### 8.2. Macrophage-Regulating Therapy

The role of macrophages in acute and chronic injury involves phagocytosis and the removal of damaged cells from the site of infection. Characteristically, M1 macrophages activate antigen-presenting cells (APCs), toll-like receptors (TLRs), and MHC-II (major histocompatibility complex) [95]. Meanwhile, M2 macrophages produce elevated levels of TGF-β and IL-10. M1 and M2 macrophages work in tandem in the migration and proliferation of fibroblasts and keratinocytes to the endothelial lining [96]. However, during impaired healing, the production of M2 macrophages is sustained, resulting in the higher expression of IL-6 and TNF-α. Herbs such as *Plectranthus amboinicus* (*PA*), which have been studied for their anti-inflammatory and antibacterial activities, have known activity against proinflammatory responses and macrophage maturation [97]. A study conducted by Lin et al. [98] on a PA-F4 extract showed homeostasis between M1 and M2 expression, which increased collagen synthesis and fibroblast proliferation. Additionally, the expression of proinflammatory cytokines (IL-1β and TNF-α) was reduced, which further manifested the macrophage and monocyte chemotaxis.

### 8.3. Stem Cell Therapy

Mesenchymal stem cells (MSCs) are one of the prominent cells that portray various benefits in graft therapies such as better healing and angiogenesis properties [99]. A study on the intradermal injection of bone-marrow-derived MSC stem cells in diabetic rats showed an increased expression of IL-10 and M2 macrophages. We highlighted the pro-healing efficiency of MSC-derived stem cells in initiating the wound healing of chronic wound ulcers by directly regulating the expression of anti-inflammatory cells such as IL-10 and M2 macrophages. The MSC-derived stem cells also showed a decreased expression of IFN-γ and TNF-α, showcasing the clearance of proinflammatory markers during the wound-healing process [100].

### 8.4. Neuropeptides

Diabetic neuropathy or acute cutaneous infection has been found to correlate with neuropeptides, which facilitate the activation of the immune system and cell proliferation. Few neuropeptides, such as calcitonin gene-related peptide (CGRP), neuropeptide Y (NPY), neurotensin (NT), and α-melanocorticotropin-releasing hormone (α-MSH) were found to be potential biomarkers in diabetic wound healing. These neuropeptides promote the expression of interferon (IFN-β), TGF-β, and the macrophage inflammatory protein (MIP-1α) [101]. A study conducted on the CGRP protein in adult epidermal keratinocytes from breast cancer showed a marked increase in the production of proinflammatory cytokines (IL-1α, IL-8, and TNF-α). This induced an increased expression of pro-nerve growth factor (NGF) and fibroblast and keratinocyte proliferation, highlighting the direct role of the neural system in skin homeostasis [102].

### 8.5. Matrix Metalloproteinase (MMP) Inhibitors

DFU infections involve the production of MMPs; these proteases are present in acute and chronic phases of infection, but their production regulates the normal healing of the wound. Normally, MMP-8 and -9 are the major inflammatory markers that are present in a DFU wound. However, MMP-8 acts as a pro-healing factor, whereas MMP-9 overexpression results in delayed wound healing [103]. A study conducted on MMP-8 knocked-out rats showed a decreased wound healing ability and lower keratinocyte movement, which ultimately increased MMP-9 expression, showcasing increased proteinase activity. This study also showed a lower expression of the anti-inflammatory cytokine TGF-β that was affected by increased MMP-9 activity [104]. Recent technologies and therapies have been developed to overcome MMP-9 overexpression. Small toxic molecules such as enantiomer racemic ND-336 [105] and ND-332 [106] have been identified as selective inhibitors of MMP-9 that result in the downregulation of proinflammatory cytokines. RNA-based therapies have also been used that involved small non-coding RNAs. A study conducted by Wang et al. [107] on microRNA (miRNA-129) showed the downregulation of MMP-9 proteinase activity, making it a possible therapeutic agent in diabetic wound healing. This study showed that miRNA-129 acts as a direct inhibitor of the MMP-9 regulatory protein (specificity protein-1; Sp-1), which in turn increases the fibroblast production and keratinocyte activation in DFU healing.

### 8.6. Monoclonal Antibodies (mAbs)

Antibody therapy or mAb therapy against infectious agents involves the determination of epitopes on the microbial flora, resulting in toxin neutralization that limits pathogen invasion. The use of mAbs against multiple surface proteins and polysaccharide regions invaded by opportunistic pathogens helps to fight various bacterial pathogens such as methicillin-resistant *S. aureus* (MRSA) and polymicrobial biofilm-forming microbes [108]. A recent report included the use of AZD6389 mAb ((anti-alpha toxin (AT), anti-clumping factor, and cross-neutralizing leukotoxin) and tyrosine kinase met (C-mAb)) against *Staphylococcus* and *Pseudomonas* infection in a diabetic rat model. The study on the AZD6389 mAb showed a decreased expression of proinflammatory cytokines (IFN-γ and IFN-α) and MMP-9 metalloproteinase with neutrophil elastase. This study also highlighted a decrease in systemic inflammation and rapid wound closure [109]. Choi et al. [110] showed that C-mAb lowers inflammatory outbursts by confirming the decreases in MMP-9 and TGF-β, resulting in fibroblast proliferation and increased vascular endothelial growth factor (VEGF) production, which improved diabetic wound healing.

### 8.7. Bioactive Molecules

The role of bioactive molecules is to speed up the angiogenesis process with beneficial endothelial vascularization, thus improving the inflammatory and antimicrobial activities in DFU healing [111]. A polyphenol-like compound; i.e., propolis, showed increased antioxidant and anti-inflammatory properties in wound healing. The topical administration of propolis showed enhanced activity against diabetic wound closure with re-epithelization via enhanced production of collagen and TGF-β signaling factors. On the contrary, there was a decrease in the expression of proinflammatory cytokines (IL-6 and TNF-α) and MMP-9 [112]. Another potent bioactive agent is naturally produced epoxy-tiglianes from bush wood trees. Epoxy-tiglianes showed an effective degradation of bacterial biofilms by inducing the production of antimicrobial genes (DEFB4 and DEF103A) and leukocyte survival factors (IL-1β, IL-17, CXCL-8, and CCL-20). It also induced protein-kinase-dependent ROS generation, which resulted in enhanced fibroblast and keratinocyte production [113]. 

The above-mentioned therapies have shown prominent effects in both acute and chronic conditions; however, major drawbacks such as limited cell survival, a shorter half-life, and adverse effects (oral ulcer and high cell death) constantly exert pressure on pharmaceutical companies to generate more sustainable therapies. Constant efforts are being made to develop more efficient therapies against DFU infections; these involve few readily available therapies such as Leucopatch [114] and Apligraph [115] as graft therapies against DFU infection. However, more drugs are still required to achieve commercialization. Most of the drugs that are in clinical trials either fail or take a long time to become commercialized; thus, the failure of most of the novel therapies is creating chaos in the current healthcare market.

## 9. Everything Starts in the Gut: Gut–Skin Axis in Non-Healing Wounds

**Figure 2:**
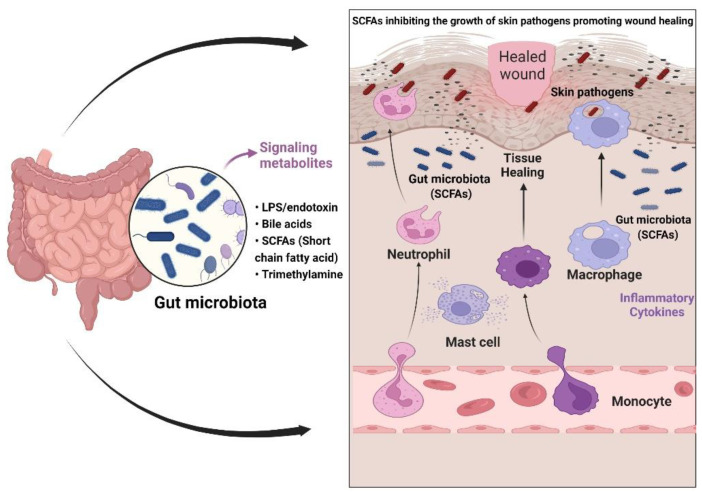
Gut–skin axis in wound healing. The gut microbiota stimulates the production of short-chain fatty acids (SCFAs) and carbohydrate metabolites that regulate the skin’s commensal bacteria. Secondary metabolites such as SCFAs regulate anti-inflammatory properties (the production of innate immune cells and cytokines), causing skin commensal bacteria to mediate effective antibacterial action against skin pathogens.

DFUs primarily involve the impaired regulation of the epidermal and endothelial layer of the peripheral foot region. The commensal or gut microbiome constantly controls the expression of growth factors (VEFG, EGF, and fibroblasts) and major skin remodeling factors (inflammatory cytokines). The epidermal keratinocytes, neutrophils, and macrophages stimulate wound healing in a coordinated manner, which ensures proper wound closure and restoring of the skin barrier [116]. The dietary pattern of an individual modulates the production of key regulators, which leads to homeostasis between the gut and the skin microbiota [117]. The efficient role of the gut is directly related to the enhanced production of innate immunity during the breakage of skin. This constant correlation between the gut and skin microbiome governs the immune-protective response that allows positive feedback for epithelial vascularization and angiogenesis [118] (Figure 2).

### 9.1. Staphylococcus epidermidis and Propionibacterium spp.: Potential Skin Commensal Probiotics

A study conducted on fiber fermentation; i.e., propionic acid and butyrate in intestinal mucosa, influenced the metabolic role of skin microbiota. For instance, *Propionibacterium* spp., which has antibacterial activity against MRSA, supported coordinated behavior between gut and skin commensal bacteria [119]. A few other microbes such as *Cutibacterium acne* and *S. epidermidis* have also shown a beneficial effect in regulating the gut–skin axis. These microbes, through their bacteriocins and antimicrobial peptides, reduce skin inflammation by inducing innate immunity and keratinocyte movement, which encourages wound healing [120]. *S. epidermidis* enhanced the expression of IFN-γ, IL-17, and the CD8^+^-associated T-cell response, enhancing the innate barrier and inhibiting the pathogen invasion [121]. Another study found a direct correlation between the gut–skin crosstalk, supporting that *S. epidermidis* is involved in fine-tuning the T-cell response via IL-1 dependent downstream signaling for maintaining tissue health during skin homeostasis [122]. Similar to the *Propionibacterium* effect, *S. epidermidis* also utilizes short-fatty acid chains (SCFAs) in demonstrating its antibacterial effect against *S. aureus* [123].

### 9.2. Lactobacillus Species: A Superior Microbiota Regulating Gut–Skin Homeostasis

*Lactobacillus* is generally one set of microbes that have shown benefits against many skin infections; it is still being used as an alternative therapy against chronic infections [124]. *Lactobacillus* has a long-known activity against chronic infections such as burn wounds and has been used effectively against wound infections. Burn wounds are a case of chronic infection that occasionally involves graft rejection, which leads to high inflammation involving the secretion of proinflammatory cytokines and plasma proteins, thereby paving the way to a high bacterial burden and chronic biofilm formation. A classic example of *L. plantarum* on third-degree-burn wound patients also showed promising results in terms of parameters such as the local allergic reaction, smoothness of the skin, and non-recovery of *Lactobacillus* after 48 h of treatment. While considering burn wounds as severe skin infections, this study also showed a differential pattern of cytokine production compared to the proinflammatory cytokines that were produced by Gram-negative and Gram-positive pathogens. The acidic pH contributed to the fibroblast migration and tissue repair, which conclusively showed the cost-effective role of *L. plantarum* [125]. A study by Poutahidis et al. on *L. reuteri* [126] showed a marked increase in the wound-healing properties via upregulation of neuropeptide oxytocin, which has a potential role in vascularization via the gut–brain axis. These studies on *Lactobacillus* highlighted the role of good gut health flora in lowering the immunomodulatory action during wound closure. 

## 10. Probiotics: Current Value in Host–Microbe Interactions

### 10.1. Probiotics: A Primitive Class of Microbes

There has been a constant debate regarding the defense mechanism of the human gut flora because bacterial colonization is too complex when it comes to beneficial or invasive microbes. A slight deviation in the microbiome equilibrium can cause serious detrimental effects on human health [127]. The relationship between gut microbial dysbiosis and diabetes is a long-standing debate that emphasizes the significant relationship between gut microbiota and glucose resistance during T2DM. According to a recent review, some gut microbes such as *Ruminococcus*, *Fusobacterium*, and *Blautia* are positively related to T2DM, while others such as *Bifidobacterium* and *Lactobacillus* are negatively related [128]. The crosstalk among various bacterial species during medical complications have always opened up the concept of “probiotics”, as they are the safest and most sustainable group of microbes that modulate positive effects in the gut–brain [129], gut–skin [130], and gut–bone [131] axes. Probiotics show a substantial positive effect on gastrointestinal metabolism and also contribute significantly to improvements in the host immune system [132]. Human studies have always defined probiotics as a strong modulator of the immune system that confer protection against numerous chronic diseases [133]. Universally, the major probiotic species are *Lactobacillus* and *Bifidobacterium*, which have numerous roles in human metabolic pathways. A study by Lim et al. [134] showed that treatment of high-fat-induced (HFD) hyperglycemic mice with *L. sakei* OK67 decreased the LPS levels in blood that was enhanced by HFD. The treatment decreased epididymal fat and suppressed the release of proinflammatory cytokines (TNF-α and IL-1) as well as the activation of NF-κB in the colon, highlighting the elevated expression of colon tight junction proteins and anti-inflammatory reactions in HFD mice. Another study on a diabetic mice model [135] showed that treatment with *L. plantarum* MTCC5690 and *L. fermentum MTCC5689* improved insulin sensitivity and decreased the expression of the intestinal tight junction’s proteins (occludin and ZO-1), which in turn elevated the circulatory levels of LPS and proinflammatory markers (TNF-α, IL-6, and monocyte chemoattractant proteins (MCP-1)), demonstrating the antidiabetic property of *Lactobacillus*. 

*Streptococcus*, *Pedi coccus*, *Enterococcus*, *Lactococcus*, and yeasts such as *Saccharomyces boulardii*, in addition to *Lactobacillus* spp., are important in regulating gut flora homeostasis [136]. Similarly, diabetes-related non-healing-wound studies have highlighted the role of commensal *Lactobacillus* spp. in the differential expression of cellular and metabolic genes that induced production of pro-healing cytokines (TNF-α, IL-12, and other non-inflammatory responses) [137]. Transcriptome profiling of *L*. *acidophilus, L. casei*, and *L. rhamnosus* showed the production of beneficial bacteriocins and secondary metabolites that can influence host metabolic regulation against various diseases [138]. In the current market, probiotics have a high yield, stability, and low-cost substrate requirement while posing no risk to the larger population [139]. Probiotics, as an effective immunomodulator, also hold great value in regulating the glycemic index of type 1 diabetes mellitus (T1DM) patients. T1DM is characterized by pancreatic beta cell damage, which results in abnormal insulin production in the regulation of blood glucose levels [140]. Continuous administration of *L. johnsonii* MH-68, *Bifidobacterium animalis* subsp. *lactis* CP-9, and *L. salivarius subsp. salicinius* AP-32 to patients with T1DM for 6 months resulted in decreased fasting blood glucose levels and HbA1c expression, as well as altered gut microbiota flora. The combination of insulin therapy and probiotic supplements in T1DM patients reduced the production of proinflammatory cytokines (IL-17, IL-18, and TNF-α), emphasizing the strong response of probiotics [141]. Another study on T2DM patients with continuous supplementation of multistrain probiotics for 12 weeks showed a decrease in glycated hemoglobin and improved the quality of life of the patients with a reduced body weight [142].

### 10.2. Basic Immunomodulatory Action of Probiotics

Probiotics have been categorized as the main factor in regulating the primary immune system and acquired immunity [143]. Probiotics generally activate dendritic cells and macrophages, which enhance the production of TGF-β, IL-4, IL-5, IL-12, and T-cell-mediated responses against pathogens [144]. The GI tract and intestinal flora are very specific for maintaining epithelial innate immunity. The presence of probiotics significantly activates humoral immune responses such as B-cell mediated immunoglobulin (Ig) production and enhances cytokine regulation. This triggers the production of anti-inflammatory cytokines (IL-10 and -12) and chemotaxis of innate immune cells (monocyte, NK cells, and dendritic cells), which induce immune homeostasis [145]. Probiotics also have a prominent role against viral or bacterial infections by modulating the NK-cell-associated T-cell response to establish antipathogenic activity [146]. A study conducted on the Gram-negative probiotic strain *E. coli* Nissle 1917 showed the production of NF-kB-mediated antibacterial peptide human β-defensin 2 (hBD-2), which promotes epithelial adhesion and activates innate cells (leukocytes and macrophages) [147]. Another study on *Bifidobacterium* showed enhanced cytokine responses and activation and maturation of dendritic cells against DSS-induced colitis. This highlighted the production of proinflammatory cytokines (IL-1β and TNF-α) that were further involved in CD4^+^ production via T-helper cells (T_H_), conferring an adaptive immune response [148].

## 11. Probiotic Effect in Acute and Chronic Biomarkers of Non-Healing DFUs

### 11.1. C-Reactive Proteins (CRPs)

**Figure 3:**
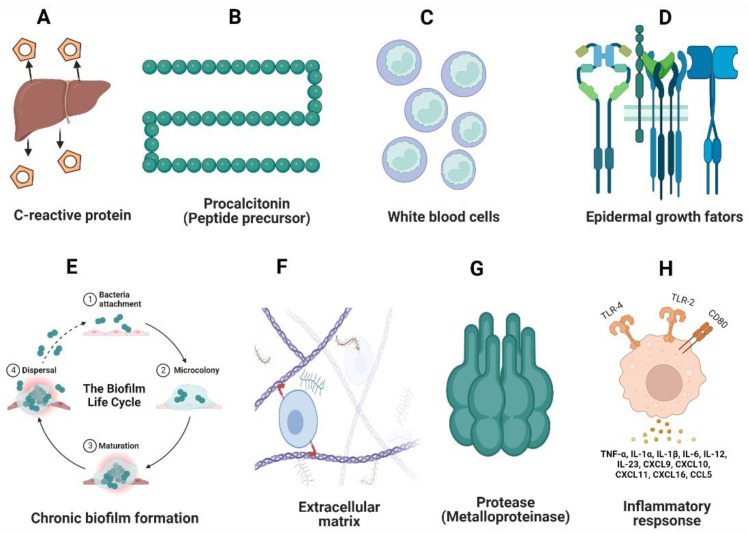
Acute (**A**–**D**) and chronic (**E**–**H**) biomarkers during DFU infections. C-reactive proteins and procalcitonin are primary proteins whose differential expression indicates the onset of DFU infections (**A**,**B**). White blood cells (WBCs) typically express information about bacterial infections (**C**). Epidermal growth factors highlight lower neutrophil expression (**D**), indicating decreased re-epithelization during wound healing. Biofilm formation by opportunistic pathogens (**E**) and reductions in collagen and the extracellular matrix (**F**) due to the decreased keratinocyte movement (**F**) occur. Matrix metalloproteinase inhibits the production of anti-inflammatory cytokines, thereby impairing wound healing (**G**). The overproduction of proinflammatory cytokines leads to a decrease in macrophage-associated proteins and T-cell-mediated immune responses (**H**).

CRPs are basic acute-phase proteins that play a role in various diabetic complications and are associated with high proinflammatory cytokines [149] (Figure 3). Currently, probiotic formulations that incorporate *Bacillus coagulans* (probiotic honey) [150] have a beneficial effect in reducing the CRP level in blood serum. Clinical trials have shown promising results of probiotics in reducing diabetic-related inflammation. For instance, we considered randomized trials on 523 participants with type 2 diabetes in which the participants were fed daily with probiotic yogurt from 2 to 12 weeks. Their inflammatory markers (CRPs, TNF-α, and IL-6) were monitored and significant decreases in the CRP level and IL-6 were found, suggesting the beneficial role of probiotics in lowering the acute phase proteins [151]. Similarly, another study conducted on patients suffering from diabetic nephropathy showed an increased production of creatinine and CRPs. These patients, who were supplemented with probiotic *Lactobacillus* and *Bifidobacterium* for 12 weeks, showed a significant reduction in the levels of CRPs, creatine, and IL-10, which highlighted the beneficial effects of probiotic supplements in reducing acute inflammation during diabetes [152]. 

### 11.2. Procalcitonin

Procalcitonin is a hormonal peptide precursor of calcitonin that shows a higher expression during the acute phase of DFUs. The level of procalcitonin rises with the increased production of proinflammatory stimuli. Generally, these peptides are associated with bacterial infections and are used as a biomarker during sepsis conditions [153]. Studies conducted on hospital-acquired DFU infections have shown an increased production of procalcitonin biomarkers. The increased biomarker level highlighted other inflammatory markers (IL-3, IL-6, and IL-8), which showed a correlation between CRPs during DFU infections [154]. Constant research has found an effective role of probiotics in lowering the production of procalcitonin with a significant decrease in IL production. A study conducted on critically ill patients with sepsis showed a high production of IL-6, protein C, and procalcitonin as major inflammatory markers during acute infections. A constant supplementation of probiotics (*Streptococcus, Bifidobacterium*, and *L. paracasei*) resulted in a drastic reduction in the serum IL-6, protein C, and procalcitonin levels after 1 week, which also highlighted the beneficial effect of probiotics against acute-phase proteins [155].

### 11.3. White Blood Cell (WBC) Count

The major risk factor apart from CRPs, procalcitonin, and serum creatine involves the WBC count during the acute stage of diabetic foot infections. An elevated level of WBCs has been significantly associated with DFUs and is significantly affected by elevated levels of blood glucose [156]. The hematological and biochemical markers during diabetic-induced osteomyelitis also include the WBC count because variation in the WBC levels estimates the degree of a diabetic foot infection [154]. As previously described, probiotics have a major effect in reducing the acute-phase proteins and inflammatory marker levels. A recent study of probiotics on non-healing distal limb infections in equine species showed a significant decrease in the WBC count after topical administration of probiotics. This significant decrease in inflammation was accompanied by a 50% decrease in the WBC count in the wound area [157]. Another study conducted on healthy individuals who were supplemented with *Lactobacillus* and *Bifidobacterium* probiotics for 6 weeks showed a decreased production of leukocytes (WBCs) and proinflammatory cytokines (IL-6 and TNF-α), showing the important role of probiotics in maintaining healthy intestinal homeostasis [158].

### 11.4. Growth Factors

The process of inflammation during diabetic neuropathy leads to an alteration in growth factors, which leads to a decrease in the chemotaxis movement of neutrophils, thus reducing the angiogenesis and endothelial growth regeneration and causing increased susceptibility to foot infections [159]. Probiotics have been shown to have a significant role in increasing VEFF-mediated cell regeneration and a major role in enhancing keratinocyte production. Studies conducted on *L. plantarum* showed low cytotoxicity and increased production of VEGF in skin regeneration and fibroblast production, demonstrating the prominent role of probiotics in the enhancement of growth factors [160]. *Lactobacillus* has also shown effects on chemokine activation and movement; these chemokines have a major role in angiogenesis and help in the recruitment of inflammatory cells [161]. 

### 11.5. Chronic Biofilm Formation

In contrast to acute wounds, chronic DFU wounds consist of 60% biofilm-forming cells, clinical growth of aerobic microbes (*P. aeruginosa* and *S. aureus*), and anaerobic microbes in the deep part of the wound; i.e., osteomyelitis, and significantly resist the entry of antimicrobial agents, which causes a delay in wound healing [162]. Conventional therapies for controlling biofilms are not very effective, hence alternatives are required, including probiotics, which have been used since the early 1990s and have shown a potential role against cutaneous skin infections. Recently, probiotic strains (*Bacillus subtilis KATMIRA* and *B. amyloliquefaciens*) showed efficient activity against wound infections [163]. Similarly, a study conducted on *L. fermentum* against potent opportunistic pathogens (*Pseudomonas* and *Staphylococcus*) showed reductions in the biofilm-forming cells and in the production of virulence factors (pyocyanin and pyoverdine) [164].

### 11.6. Extracellular Matrix (ECM)

Normal wound healing involves collagen remodeling and degradation of fibril proteins by MMPs, but a DFU condition tends to overexpress the fibrils, resulting in distortions in ECM remodeling. Fluctuations in the levels of collagen and fibrinogen in blood plasma decreases elastin and fibronectin production in the tissue remodeling [165]. The damaged host ECM results in the movement of opportunistic pathogens by lowering the ECM binding and promoting virulence. Some probiotic species have shown an effective role in reinitiating production of collagen I and fibronectin [166]. A study conducted on *L. casei* showed its significant effects in ECM remodeling and fibronectin production by revealing the efficient movement of fibroblasts and keratinocytes for re-epithelization [167].

### 11.7. Matrix Metalloproteinase (MMP)

During delays in the wound-healing process, MMP-9 plays a role in the production of acute-phase proteins and proinflammatory cytokines. The imbalance between the ECM formation and high levels of MMP-2 and MMP-9 in macrophages inhibits the expression of anti-inflammatory factors such as TimP-1 and TimP-2, leading to healing dysbiosis [168]. Most studies have not shown the regulation of probiotic species in these MMP proteins during DFUs, but some studies on sepsis showed a significant decrease in MMP when *Lactobacillus* spp. was administered. A study conducted by Maghsood et al. [169] on cell-free supernatants (CFS) of *L. acidophilus* and *L. rhamnosus* showed a decreased expression of MMP-9 in human monocyte cells. This highlighted a significant increase in the cell-surface expression of CD147 and TimP-1 and TimP-2, which promoted anti-inflammatory properties. Another study on an ethanol extract from *L. plantarum* showed a significant decrease in MMP-9, IL-6, and TNF-α expression in streptozotocin-induced diabetic rats. The study showed the production of collagen fiber and extracellular matrix, showcasing wound healing and the positive effect of *Lactobacillus* in lowering the inflammatory response [170].

### 11.8. Proinflammatory Cytokine Response

The infiltration of cytokines is the major factor related to increased inflammation during a chronic DFU condition. The major process of healing is retarded by excessive proinflammatory cytokine secretion, macrophage polarization, tissue destruction, loss of angiogenesis, and bacteria-induced inflammation [171]. To overcome the immunomodulatory burst, probiotics have been constantly recognized as an efficient therapy. Various studies showed the downregulation of inflammatory pathways. For instance, *L. reuteri* extracts increased the expression of the inflammatory protein MMP-1 with downregulation of TGF-β signaling. This enhanced the proliferation of skin fibroblasts. In addition, *L. reuteri* extracts increased the expression of P13-AKT pathways, which play a prominent role in cell differentiation and apoptosis [172]. Another clinical study of DFU patients that used topical administration of *L. plantarum* showed a remarkable difference in terms of angiogenesis and vascular area of the wounds. In addition, the study showed M1 and M2 macrophage polarization in fibroblast proliferation and tissue remodeling [173].

## 12. Results

The search yielded 17 potentially relevant articles related to the role of probiotics in modulating acute and chronic markers of DFUs, 4 of which were randomized control trials, placebo trials, and single blinded multicenter studies. The randomized trial studies used probiotic strains such as *L. acidophilus*, *Bifidobacterium*, *L. reuteri*, and *Streptococcus* spp. to lower the expression of acute-phase markers such as C-reactive proteins and procalcitonin. In a clinical trial study, *L. plantarum* and *Bifidobacterium* reduced the white blood cell (WBC) count and proinflammatory markers, highlighting the efficacy of probiotic supplements. One systematic meta-analysis review also highlighted the role of yogurt consumption in lowering the acute-phase proteins by reducing the expression of IL-6, a proinflammatory marker. The remaining 11 articles involved in vivo and in vitro observational studies. The major probiotic studies related to acute and chronic conditions are listed in Table 2.

## 13. Novel Prospects of Probiotic-Related Therapies against DFUs

Currently, few probiotic therapies have shown an efficient response against non-healing wound ulcers (diabetic and non-diabetic). Non-healing diabetic wounds showed a decreased expression of vascularization due to reduced angiogenesis and insulin metabolism. The few probiotics mentioned in Table 3 highlight the efficient response against intrinsic mediators of non-healing diabetes-associated foot ulcers. 

Apart from the above-mentioned probiotic supplements (Table 2), few novel avenues can be utilized to increase the efficiency pf probiotic strains against DFU infections. Some of the novel prospects are highlighted below. 

### 13.1. Probiotic Encapsulation

To attain high efficiency at the wound site, probiotics need to be biocompatible and non-toxic. The heterogeneous nature of chronic wounds involves polymicrobial flora that constantly trigger the immunogenic responses. The fine biocompatibility and non-toxicity of probiotics can be maintained by using various encapsulating agents such as polymeric substances (e.g., chitosan, alginate, and hydrogels). These agents have proven non-toxicity and an environmentally friendly nature that could protect probiotics from a harmful pathogenic environment [179]. A recent study conducted on probiotic encapsulation with antibiotics in an alginate-mediated encapsulating agent showed an increased potency of the tobramycin antibiotic in the eradication of MRSA strains [180]. Another study on delivering probiotics found that a potent self-assembling coated system composed of tannic acid and poloxamer showed strong intestinal adhesion, ROS production, and higher deleterious effects against DSS-induced-colitis mice [181]. Similarly, heparin-based hydrogels also showed a potent effect against inflammatory markers of wound healing by regulating M1 and M2 macrophage polarization [182] and reducing the expression of proinflammatory markers (IL-1β, TNF-α, and NF-kB). These potent regenerative concepts can be applied to the chronic DFU situation via co-administration of empirical antibiotics with a probiotic in an enclosed shell, which will limit the effects of harmful bacteria on probiotics and will also provide the benefit of targeted therapy against polymicrobial infection.

### 13.2. Prebiotics and Synbiotics: Nanoformulations

Prebiotics is a well-known concept in which the intake of dietary fiber invokes gut microbes to produce beneficial factors, while synbiotics is a concept in which both a probiotic and prebiotic are combined [183]. Both concepts involve the positive regulation of the host microbiota in that they showed beneficial effects in regulating intestinal homeostasis. A study with *Lycium barbarum* (herb)-derived polysaccharides on mice fecal microbiota showed a significant increase in the growth of *Lactobacillus* and *Bifidobacterium*, thereby modulating the intestinal microbial communities. Moreover, the inflammatory response was also varied significantly by changing IL-6 and TNF-β production [184]. Synbiotic studies involving the use of probiotic *Bacillus coagulans* [185] and a prebiotic grape extract with a *Lactobacillus* mixture [186] showed prominently decreased inflammatory markers in caco2 cells and decreased inflammation in diabetic patients. Non-digestible carbohydrate pullulan used as an NP-like prebiotic supplement with *L. plantarum* showed increased production of antimicrobial peptide that could eradicate Gram-negative pathogens [187]. The potency of nanotherapies with prebiotics and synbiotics can be applied to DFU infections because the gut microbiota play a major role in modulating skin commensal bacteria to produce antimicrobial peptides against pathogens. Therefore, various non-digested fibers should be screened and used as sources for nanoformulations with probiotic supplements to modulate the production of the inflammatory response during chronic conditions.

### 13.3. Probiotic-Derived Biogenic Nanoparticles (NPs)

**Figure 4:**
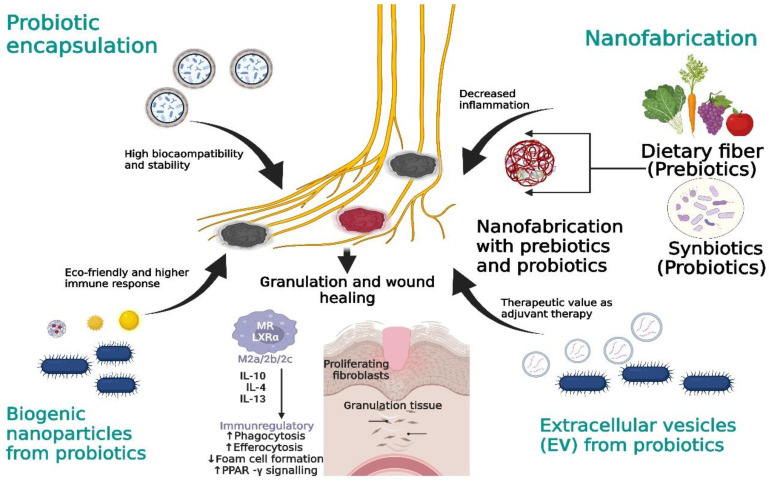
Novel approaches to target DFU prognosis. Probiotic encapsulation ensures the probiotics’ long-term stability against chronic DFUs. Dietary fiber will be used in nanofabrication with prebiotics and probiotics for efficient probiotic regulation. Biogenic NPs boost immune activation; probiotic-derived EVs can be used as an alternative therapeutic delivery agent.

Biogenic materials involve plants, microbes, and fungi; currently, these microbes are being used as a potent source to produce NPs. These NPs provide an edge over conventional NP synthesis due to their lower toxicity and high durability against various medical treatments [188]. Microbes are considered to be capable producers of NPs using elements such as silver (Ag), selenium (Se), gold (Au), and copper (Cu), which have enhanced therapeutic values. To this end, environmentally friendly and economic probiotics such as *L. paraplantarum* and *L. crustorum*, *L. delbrukii*, and *L. plantarum* are extensively being used to treat various pathological conditions (antibacterial, antioxidant, or even chronic biofilm conditions) [189]. The use of biogenic NPs using probiotic microbes showed a beneficial effect on chronic DFU infections because the above NPs could strongly produce ROS that efficiently degraded cell walls, could be synergistically used with other antimicrobial compounds to enhance immune cell activation, and could act as an anti-biofilm agent. NPs can overcome the limits of chronic ulcer biofilm, which makes it difficult for antimicrobial compounds to enter the wound site and results in a delay in wound healing (Figure 4).

### 13.4. Probiotic-Derived Extracellular Vesicles (EVs)

Another class of nanotherapeutics involves extracellular vesicles (EVs), which are the membrane vesicles that can carry biogenic compounds including lipids, proteins, and nucleic acids. These vesicles have recently gained much value due to their intrinsic antimicrobial activity and their use as a successful delivery agent against various chronic illnesses [190]. A study using EVs derived from *Propionibacterium freundenreichii* showed enhanced immunogenic activity in HT-29 human epithelial cells. In this study, NF-kB and IL-8 were drastically reduced as compared to the non-treated cells with EVs, which supported the anti-inflammatory response of EVs against chronic illness [191]. Other studies of probiotic-derived EVs showed their potential role as adjuvants [192] in modulating the proliferation of epithelial growth factors during DSS-induced colitis [193]. Based on the above results, it is obvious that probiotic-derived vesicles have a potential role in reducing acute and chronic inflammation, since most of the inflammatory cytokines and biomarkers of IL-8, NF-kB, and TNF-α are also the prominent markers in DFU infections. Therefore, EVs derived from probiotics can be utilized as a futuristic therapeutic material in the betterment of the healing prognosis of DFU wounds.

## 14. Management of Acute and Chronic DFU Conditions via Probiotic Remodeling

The fundamental issue behind the global glycemic index is the lifelong risk of acquiring lower-limb DFUs. The current situation is disastrous because lower limbs are either amputated or patients suffer from emotional and physical stress as a result of exorbitant healthcare costs. Foot deformity due to a DFU is a progressive disorder that transforms the disorder into a life-threatening risk in due course. The ongoing quest for efficient technologies or therapies to assess DFU wounds has shown a chronicity of such wounds that encompasses a variety of pathological problems that require effective care [194]. Loss of sensation or persistent pressure in the limb region requires adequate foot care management as well as effective therapies to combat the resulting issues with a non-healing wound. The margin between the acute and chronic situations in a DFU is so narrow that even a subcutaneous hemorrhage or simple crack in the foot might result in a chronic gangrene situation. The complexity of DFU prognosis often requires proper medical practices such as ulcer classification or grading. The evaluation system provides both sufficient information to clinicians and comprehensive knowledge regarding the entire history of the foot complications of patients [195]. Sufficient data from the wound morphology provide evidence that may be utilized when prescribing medical facilities to the patient.

Acute and chronic diseases are generally discrete phases that entail the control of numerous parameters such as the blood serum level, biomarker presence, and inflammatory cytokines. The acute stage is characterized by the normal degradation of growth factors, which deregulate the vascularization process in normal healing. This causes systemic inflammatory responses that involve macrophages and neutrophils, resulting in decreased production of anti-inflammatory cytokines and constant tissue hypoxia in the peripheral wound region. Normal or acute healing takes 2 to 3 months and includes cell mitosis and tissue granularization, but constant and recurrent foot trauma results in a chronic condition of the foot region that includes a high inflammatory response involving an innate immune response and stimulation of proinflammatory cytokines. This makes the foot region more susceptible to infections. The high glycemic index also causes vascular neuropathy, which in turn causes a lack of angiogenesis, making the foot region more susceptible to persistent infections, which due to the formation of biofilm micro-communities in later stages, slows down the wound-healing process by more than 8 to 10 months. Out of all the factors mentioned here, the immune response plays a vital role in a significant delay of the wound-healing process because it is a host’s immune response that generally activates the proinflammatory modulators that cause immunogenic bursts at the site of infection.

The dysregulated response during DFUs involves the production of acute-phase proteins, WBCs, and other serum proteins that provide the primary signs of infection. Then, inflammatory cytokines come into the picture that involve the significant upregulation and downregulation of immunoregulators. Chronic DFUs primarily involve the dysregulation of neutrophils and macrophages, which initiates the downregulation of dendritic cells (MCP-1 and IFN-γ) and MMP-9. The imbalance in macrophages causes a decrease in the growth factors (VEGF and EGF) and tissue remodeling of collagen and fibronectin. This regulates the movement of proinflammatory cytokines (IL-1, IL-1β, and TGF-α), which increases the infection rate of the region and dysregulates the T-cells’ maturation. The cell-mediated immunity is hampered by a decreased, restricted movement of T-regulatory cells (Treg) and T_H_ to the site of the infection. Constant production of chemokines and cytokines at the site of the wound lowers the epithelial remodeling and diminishes the vascularization property of keratinocytes, thereby rupturing the endothelial lining, which certainly paves the way for pathogens to enter. These pathogens secrete virulence factors that inhibit the normal healing process of the wound and involve the prognosis of tissue necrosis and a low oxygen state at the site of infection, causing it to reach the gangrenous state or the amputation stage.

Regarding the management of wound ulcer situations, various novel therapies that directly target the immune responses during DFU infections, such as therapies using fibroblasts, macrophage activation, stem cell or MMP inhibitors, and mAbs, have shown a drastic reduction in the proinflammatory mediators but have not been efficient in terms of longevity and static efficient responses. The major drawbacks involve a shorter half-life, repeated administration, degradation via a proteolytic environment of the wound, high cell death, expensiveness, hydrolysis at a low pH, limited downstream processing, renal clearance, and a high production cost. Still, there are FDA-approved drugs that have shown potent activity in lowering inflammatory responses, but there should be a continuous search for more efficient and better therapies that can regulate the host metabolism by lowering chronic malignancies. In the quest to find efficient therapies, researchers found a constant relationship between the microbe and the host cell response. However, a decade has passed and there have been a substantial amount of data that showed the influence of environmental microflora on human health; several microbes have been identified as pathogens, including basic categories such as *Staphylococcus*, *Streptococcus*, *Bacillus*, *Pseudomonas*, *Klebsiella*, and other fungal bodies. There is one class that has always had a beneficial role in host–microbe interactions; i.e., “probiotics.” The constant regulation between the host gut microbiota and skin has shown a consistent homeostasis relationship between the dermal and mucosal microbiota. The regulation of various metabolites (short-chain fatty acids, exopolysaccharides, lipids, and other metabolites) directly helps to maintain the protective microbiota in cutaneous and subcutaneous layers against pathogenic interactions.

Probiotics have been known to provide a protective barrier against various pathogenic species. These microbes constantly regulate the gut–skin metabolism to provide essential metabolites such as bacteriocins and anti-inflammatory molecules, which modulate the innate immune response against DFUs. Most probiotics degrade the proinflammatory chemokines (CXCL10), which have a role in stimulating inflammatory cells. Some species have been found to regulate the epithelial lining by regulating protein kinase activities. *L. plantarum* provided a maximum benefit when used in a probiotic cocktail with *Bifidobacterium* because it regulated immunoglobulin activity by establishing a sustainable relationship with the tissue repair [196]. In the current scenario, probiotic drinks have shown numerous benefits in regulating all three primary immune metabolisms; i.e., innate, adaptive, and acquired immunity, by constantly regulating the leukocyte, macrophage, and neutrophil chemotaxis [197]. A delay in wound healing is a chronic condition that is affected not only by neuropathy or vasculopathy, but also by the extent of infection. The high bacterial density at the DFU site plays an important role in forming biofilm communities. Constant intercommunicating signals (quorum sensing and siderophore production) increase the chances of forming compact microbiota, which often results in hypoxia situations and reduced blood flow, ultimately forming gangrene. These pathogens act as natural barriers against antimicrobial compounds and evade the host’s natural immune mechanisms by eradicating the surrounding tissue, which leads to inflammation and a delay in the wound-healing process. Therefore, these tandem roles of the host innate response and chronic stimulation increase the chances of amputations or long-term infections. Therapies have shown effectivity to a great extent, but consistent deterioration of therapies might complicate the treatment efficiency; therefore, probiotics or sustainable dietary intake will not only moderately affect the glycemia index (diabetes), but also will regulate the innate immune response by limiting other risk factors such as neuropathy, nephropathy, or retinopathy. Because most of the inflammatory cytokines or proinflammatory markers have a prominent role in maintaining the gut flora metabolism in normal wound-healing procedures, the same immunomodulators can have a detrimental effect during chronic dysbiosis.

## 15. Limitations

The use of entire microbes as probiotics has beneficial effects in reducing systemic inflammation by maintaining gut–skin–brain homeostasis. However, effectively defining the microbiome as a “probiotic” still requires an extensive number of supporting explanations in terms of its use in a larger population and clinical outcomes. Currently, trial-and-error-based strategies are being explored; therefore, medical interventions, more detailed analyses, and proper clinical trials are required to generate more user-friendly data with conclusive knowledge related to dosage values. Normally, the source of probiotics varies from Gram-positive to Gram-negative bacteria, and environmental microbes acclimatize themselves according to the respective niches. Therefore, in some instances, probiotic ingestion might allow probiotic species to reshuffle their entire genetic makeup, which can cause detrimental effects on patients; the impact on critically immunocompromised patients would be especially severe. Apart from this, in the era of antibiotic resistance, parameters such as drug usage, the potentiality of drugs, and the prescription of drugs ultimately influence the microbial flora of the human system. The major problems of these probiotic species from friend to foe involve horizontal gene transfer mechanisms; such mechanisms would limit the use of probiotic products. Certain examples such as *S. epidermidis* and *Propionibacterium* spp. have shown numerous benefits in terms of the production of secondary metabolites and fatty acids, which constantly limit the growth of other dermal pathogens. However, a slight imbalance in the gut–skin axis may cause them to become detrimental to the host’s microflora.

## 16. Conclusions

### 16.1. Current Progress

Prebiotics and synbiotics are important in regulating an individual’s intestinal mucosa; products combining dietary fibers with probiotics have been shown to modulate intestinal gut flora, which in turn can reduce the glycemic index and lipid metabolism [198]. The commercialized probiotic product, which includes a cocktail of *Lactobacillus* spp. and *Bifidobacterium* spp. in a capsule, provides nearly all of the benefits in modulating an effective immune response against chronic DFU infections. A novel concept known as “postbiotics” is currently being investigated as a replacement for probiotics. Postbiotics are metabolic products released by probiotic organisms that have several advantages, including a high scale-up, prolonged storage viability, and potent immunomodulatory properties. Similarly, a concept known as “paraprobiotics”, which involves dead or inactive probiotic cells, has demonstrated beneficial anti-inflammatory and anti-biofilm properties, as well as an improved gut barrier function and immunomodulatory properties, suggesting their use in reducing chronic wound infections [199]. These concepts may lead to effective gut–skin homeostasis. However, large-scale clinical trials are required before they can be effectively rolled out in the market.

### 16.2. Future Perspectives

There is a need for metagenomic approaches to identify as many probiotics or beneficial microbes as possible because such microbes have the potency to induce our intestinal homeostasis. The DFU is a severe metabolic disorder that is combined with dysbiosis of the neural system (motor neuropathy and vascular neuropathy); its subsequent progression leads to a chronic situation of the wound. Probiotics generally have much potential in regulating wounds at the acute and chronic stages. Therefore, commercially available or newly discovered beneficial microbes can be used as resources for bioactive materials such as biogenic molecules or EVs to be combined with DFU-efficient NPs, which can be alternatively used with hydrogel dressings or can be used as a synergistic molecule with antibiotics in biofilm-related DFUs. Finally, this review on probiotics represents an interesting area of research in treating the acute and chronic conditions of DFUs and provides an accurate assessment of the manner in which they regulate gut–skin homeostasis and how current advancements in prebiotics and synbiotics can increase the potency of probiotics against DFU infections.

## Figures and Tables

**Table 1:** Advantages and disadvantages of conventional therapies.

Therapy	Advantages	Disadvantages	Reference
Debridement	- Release of foot pressure - Rapid healing and wound re-epithelization	- Local irritation and inflammation - Bleeding and intense pain	[86]
Hyperbaric oxygen therapy	- Adequate blood supply - Increased angiogenesis	- Continuous use causes O_2_ posing - Lung and sinus damage	[87,88]
Shock wave therapy	- Promotes angiogenesis - Fibroblast proliferation	- Cost-ineffective - Inefficient in acute neuropathy	[89]
Offloading therapy	- Healing of neuropathic forefoot and midfoot - Release of pressure	- Lack of standardization - No proper pressure management	[90]
Larval therapy	- Lower bacterial load	- Amputation - Infection and swelling	[91]
Antibiotic treatment	- Multiple actions - Broad-spectrum effects	- Drug resistance - ower activity against biofilm - Hypersensitivity	[92]

**Table 2:** Features of probiotic studies associated with acute and chronic conditions.

Author	Acute/Chronic Markers	Study Description	Probiotic Source	Level of Proteins or Acute and Chronic Marker Response	Microbial Burden	Inflammatory Mediators
Mousavi et al. [151]	CRP	Systematic meta-analysis	Probiotic yogurt	<3 mg·dL^−1^	NA	IL-6
Mafi et al. [152]		Randomized placebo trial	*Bifidobacterium*, *L. acidophilus*, *L. fermentum*, and *L. reuteri*	3.8 ± 1.9 (mg·L^−1^)	NA	IL-1, TNF- α, and TGF-β
Sanaie et al. [155]	Procalcitonin	Randomized placebo trial	*Bifidobacterium*, *Lactobacillus*, and *Streptococcus*	1.67 ± 1.27 (μg·mL^−1^) ~0.47 ± 0.41 (μg·mL^−1^)	NA	IL-6 and acute phase proteins
Stene et al. [158]	WBC	Clinical trial	*Bifidobacterium infantis* and *L. plantarum*	10^9^ circulating leukocytes	NA	IL-6 and IL-10
Varma et al. [164]	EPS	Observational research analysis	*L. fermentum*	2.5 and 5 μg of culture supernatant: antibiofilm activity	*Pseudomonas* and *Staphylococcus* biofilm	NA
Lorca et al. [166]	Extracellular matrix, collagen, fibronectin	Observational research analysis	*L. acidophilus*	Improved collagen and fibronectin binding (4.6- to 6.3-fold)	NA	NA
Chuang et al. [170]	MMP-9	Observational research analysis	*L. plantarum*	Low MMP-9	NA	Low levels of IL-6 and TNF-α
Han et al. [172]	Proinflammatory cytokines	Observational research analysis	*L. reuteri*	Mesenchymal stem cell migration	NA	Enhanced MMP proteinase, TGF-1
Arganaraz et al. [173]	Innate immune response	Observational research analysis	Subcutaneous debridement plus *Lactobacillus* (topical)	Enhanced phagocytosis and macrophage maturation	Lower bioburden	M1 and M2 macrophages

Note: NA indicates that data were not available.

**Table 3:** Probiotic responses against DFU infections.

Source	Response against DFU Markers	Reference
*L. rhamnosus* GG lysate	Chemokine movement (CXCL2 and CXCR2) induced re-epithelization and keratinocyte movement during non-healing wound	[174]
*Bifidobacterium bifidum*, *L. acidophilus*, *L. casei*, and *L. fermentum*	Decrease in total cholesterol level and high sensitivity CRPs in DFU patients after 12 weeks of continuous supplementation of probiotics	[175]
Genetically modified *L. reuteri* with a plasmid-encoding CXCL2 chemokine	Rapid wound closure with persistent proliferation of dermal cells and prolonged bioavailability of immune cells such as macrophages	[176]
Ethanol extract from *L. plantarum* TWK10	Enhance wound-healing properties with reduced expression of proinflammatory markers (TNF-α, IL-6, and MMP-9)	[170]
*Bifidobacterium lactis*, *L. acidophilus*, *L. paracasei*, and *L. rhamnosus*	Higher neovascular formation and reduced expression of proinflammatory markers	[177]
*L. bulgaricus* and *L. plantarum*	Decreased expression of proinflammatory markers (IL-1β and TNF-α); increased expression of anti-inflammatory markers (IL-10 and TGF-β)	[178]

## Data Availability

Not applicable.
